# MIB2‐Mediated SUZ12 Ubiquitination Regulates Clonal Proliferation in Patients With Paroxysmal Nocturnal Haemoglobinuria

**DOI:** 10.1111/jcmm.70597

**Published:** 2025-06-06

**Authors:** Hui Liu, Lijie Zeng, Chaomeng Wang, Yingying Chen, Liyan Li, Zhaoyun Liu, Honglei Wang, Rong Fu

**Affiliations:** ^1^ Department of Hematology Tianjin Medical University General Hospital Tianjin China; ^2^ Tianjin Key Laboratory of Bone Marrow Failure and Malignant Hemopoietic Clone Control Tianjin China; ^3^ Tianjin Institute of Hematology Tianjin China

**Keywords:** MIB2, paroxysmal nocturnal haemoglobinuria, PRC2 complex, proliferation, SUZ12

## Abstract

Secondary gene mutations are one of the main mechanisms underlying paroxysmal nocturnal haemoglobinuria (PNH) clone proliferation. Our previous studies showed that SUZ12 participates in PNH clone proliferation by regulating H3K27me3. However, the mechanism underlying the SUZ12 function remains unclear. Immunoprecipitation and mass spectrometry were used to identify SUZ12 interacting proteins in *PIGA*‐KO K562 cells. Furthermore, RNA‐seq was used to explore the signalling pathways. Finally, colony formation assays, western blotting, and flow cytometry were performed to determine the proliferative ability of the cells. We identified 59 potential proteins that interact with SUZ12 and revealed a physical interaction between MIB2 and SUZ12 exclusively in the K562‐KO cell line. Furthermore, we emphasise the vital involvement of the MIB/HERC and ZZ‐type domains in the physical association between MIB2 and SUZ12. After MIB2 knockdown, SUZ12 protein decreased while the ubiquitination levels of SUZ12 were enhanced. Additionally, PRC2‐related target genes were upregulated in the siMIB2 group. SUZ12 and H3K27me3 expression levels and cell proliferation significantly decreased after MIB2 knockdown, whereas cell apoptosis significantly increased. MIB2 protein levels are also elevated in patients with PNH. In conclusion, MIB2 affects the stability of the PRC2 complex by mediating SUZ12 ubiquitination, which in turn regulates PNH clone proliferation.

## 1 Introduction

Paroxysmal nocturnal haemoglobinuria (PNH) is a rare clonal disorder of haematopoietic stem cells, characterised by hemolytic anaemia, thrombosis, smooth muscle dystonia, and bone marrow failure [1]. PNH arises from the clonal expansion of haematopoietic stem cells carrying somatic mutations in the PIG‐A gene, which plays a vital role in the synthesis of glycosylphosphatidylinositol (GPI) anchors. These anchors are necessary for attaching various proteins, including the complement regulatory proteins CD55 and CD59, to the cell surface [2]. In PNH, mutated stem cells give rise to erythrocytes, leukocytes, and platelets lacking GPI‐anchored proteins, rendering them susceptible to lysis mediated by the complement system. It is the selective advantage (due to genes or immune escape) that allows the PIG‐A mutant clone to expand, resulting in the emergence of a population of cells deficient in GPI‐anchored proteins [3, 4]. Such clonal expansion can arise from a single haematopoietic stem cell or involve multiple stem cells, leading to a heterogeneous population of cells with varying levels of GPI‐anchored proteins [5, 6].

We previously showed that SUZ12 protein expression is elevated in CD59^−^ cells from PNH patients compared to CD59^+^ cells and normal controls. A positive correlation was observed between SUZ12 protein and H3K27me3 expression. Furthermore, there is a positive correlation between the protein expression level of SUZ12 in peripheral blood CD59^−^cells and the proportion of abnormal clones in patients with PNH. In addition, the knockdown of SUZ12 in THP‐1 cells with PIGA knockdown led to decreased H3K27me3 levels, impaired cell proliferation, increased apoptosis, and cell cycle arrest in the G0/G1 phase [7]. These findings indicate that the upregulated expression of SUZ12 in abnormal clone cells of patients with PNH, along with its modulation of histone H3K27me3 levels, exerts regulatory effects on cell proliferation, apoptosis, and the cell cycle, thereby facilitating the clonal expansion of abnormal PNH clones.

In this study, we investigated the potential role of specific proteins in PIGA knockout cells in regulating the function of the PRC2 complex by modulating SUZ12 protein alterations, consequently affecting cell proliferation. Previous reports have documented ubiquitination modification sites on SUZ12 and revealed that it is regulated by the ubiquitin‐proteasome system as a substrate of USP3 [8]. Mass spectrometry following SUZ12 protein purification revealed MIB2 as an interacting E3 ubiquitin ligase, warranting further investigation. We further investigated whether MIB2 facilitates SUZ12 ubiquitination, thereby modulating the function of the PRC2 complex and regulating clonal expansion in PNH. Our findings would help in the identification of potential therapeutic targets for PNH.

## 2 Materials and Methods

### 2.1 Patients

This study included 15 patients diagnosed with classic PNH who were admitted to the Haematology Department of Tianjin Medical University General Hospital between October 2021 and December 2022. Classic PNH was diagnosed according to reference standards in the literature [9]. All participants provided informed consent before participating in the study, which was approved by the Ethics Committee of Tianjin Medical University.

### 2.2 Cell Culture and Transfection

We constructed a *PIGA* knockout K562 (K562‐KO) cell line using CRISPR‐Cas9 [6]. K562‐WT/KO cells were cultured in RPMI‐1640 medium supplemented with 10% foetal bovine serum (FBS). For RNA studies, K562 cells were transfected with the predesigned small interfering RNAs (siRNAs) at a final concentration of 25 nM. The cells were transfected with siRNA duplexes using RNAiMAX according to the manufacturer's instructions. For the plasmid transfection assay, K562 cells were transfected with plasmid using Lipo3000 according to the manufacturer's instructions. The effect of gene knockdown or overexpression was evaluated by western blotting at 48 h post‐transfection. SiMIB2 kit, pcDNA3.1‐MIB2‐his (includinge FL, D1, D2, D3, and △D) was purchased from GENEWIZ (Suzhou, China). pCMV6‐SUZ12‐flag was purchased from Origene (CA, USA).

### 2.3 Cell Growth Assays

Cell growth was assessed using a cell cycle kit according to the manufacturer's instructions. For the colony formation assay, transfected cells were seeded in 6‐well plates at 3000 cells per well. Two weeks later, the colonies were stained overnight with 0.1% NBT. The number of colonies with diameters > 1.5 mm was counted.

### 2.4 Mass Spectrometric Analysis

LC–MS/MS analysis was performed using a Q Exactive mass spectrometer (Thermo Scientific) coupled to an Easy nLC (Proxeon Biosystems, now Thermo Fisher Scientific) for 60 min. The mass spectrometer was operated in the positive ion mode. MS data were acquired using a data‐dependent top20 method dynamically choosing the most abundant precursor ions from the survey scan (300–1800 m/z) for HCD fragmentation. The automatic gain control (AGC) target was set to 1e6, the maximum injection time was set to 50 ms, and the number of scan range was set to 1. The dynamic exclusion duration was 30.0 s. Survey scans were acquired at a resolution of 70,000 at m/z 100, and the resolution for HCD spectra was set to 17,500 at m/z 100; the automatic gain control (AGC) target was set to 1e5, isolation width was 1.5 m/z, microscans were set to 1, and the maximum injection time was set to 50 ms. The normalised collision energy was 27 eV, and the underfill ratio, which specifies the minimum percentage of the target value likely to be reached at maximum fill time, was defined as 0.1%. The instrument was run with peptide recognition mode.

### 2.5 Western Blot

Whole‐cell lysates were harvested from the treated cells and resuspended in a 5 × SDS‐PAGE loading buffer. The boiled protein samples were separated using SDS‐polyacrylamide gel electrophoresis and transferred onto polyvinylidene fluoride membranes. Membranes were incubated with primary antibodies (Cell Signaling Technology, Danvers, MA, USA) followed by HRP‐conjugated secondary antibodies (Cell Signaling Technology), and bands were visualised using an enhanced chemiluminescence system.

### 2.6 Flow Cytometry

Cells were stained with cell surface markers for 15 min, washed with PBS, and resuspended. Intracellular antibodies (BioLegend, San Diego, CA, USA) were used for staining after permeabilisation of the cell membranes. The cells were detected using a Beckman Coulter instrument. Data was analysed using FlowJo software.

### 2.7 Statistical Analysis

Data were plotted using GraphPad Prism 8.0. Values were expressed as mean ± SD. Statistical significance between the two groups was determined using an unpaired two‐tailed Student's t‐test. Comparisons of multiple groups were conducted using two‐way ANOVA with Sidak's multiple comparison test (SPSS, version 17.0). The criterion for statistical significance was set at a *p* value less than 0.05. The raw MS data for each sample were searched using the MASCOT engine (Matrix Science, London, UK; version 2.2) embedded in Proteome Discoverer 1.4 software for identification and quantitation analysis 3.

## 3 Results

### 3.1 Correlation Analysis of SUZ12 Expression Levels and Clinical Indicators of PNH

**FIGURE 1:**
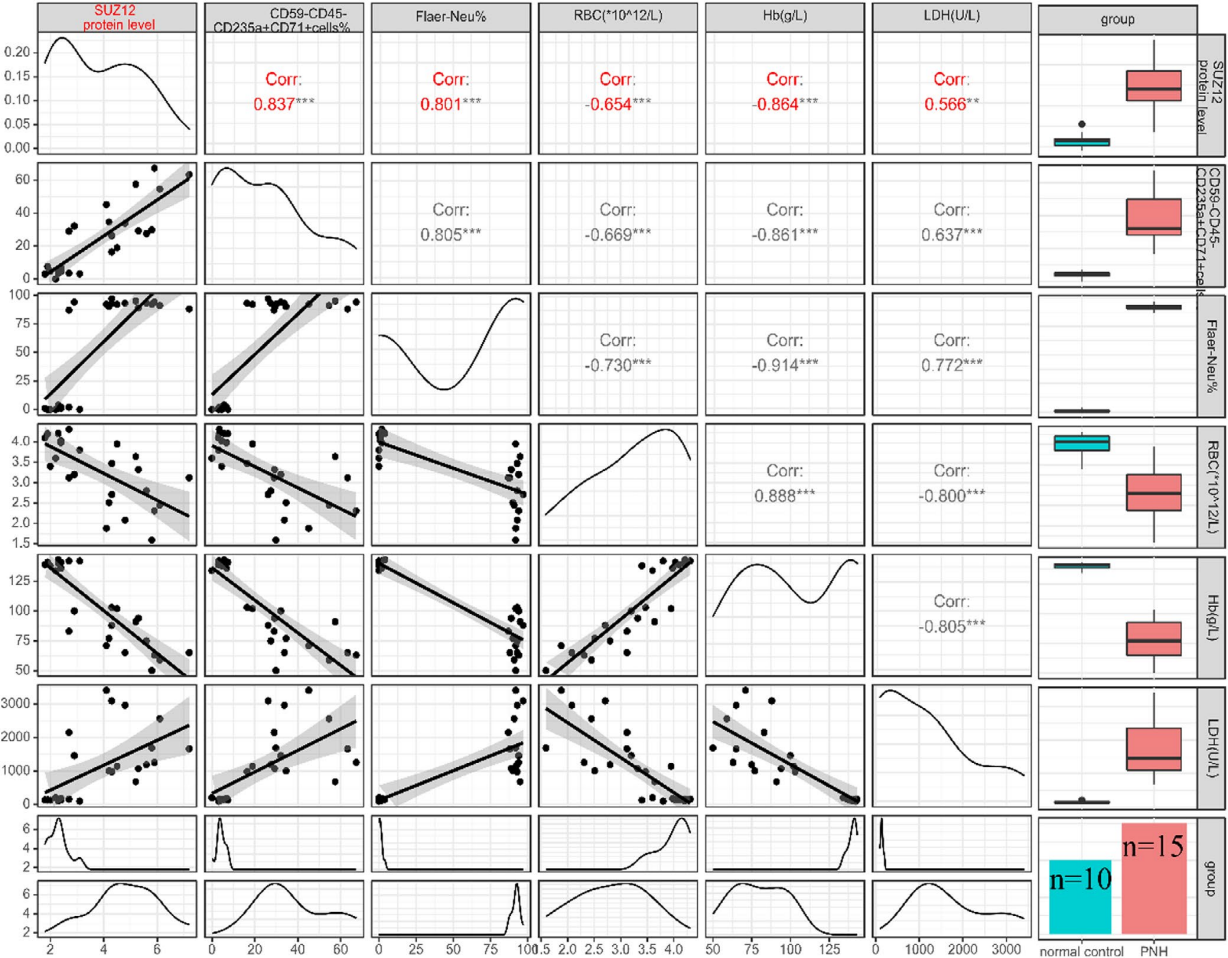
Correlation analysis of SUZ12 protein expression level and clinical indicators in PNH patients. The expression level of B4GALT5 mRNA is significantly negatively correlated with the proportion of Flaer negative nucleated red blood cells (*r* = −0.731, *p* < 0.001) and LDH levels (*r* = −0.701, *p* < 0.001), while it is significantly positively correlated with Hb (*r* = 0.878, *p* < 0.001). ***: *p* < 0.001.

We evaluated the correlation between SUZ12 protein expression and clinical indicators in patients with PNH (Table 1). The results showed a significant positive correlation between SUZ12 protein expression and the proportion of CD59‐nucleated red blood cells (*r* = 0.837, *p* < 0.001) and LDH levels (*r* = 0.566, *p* < 0.001). In contrast, a significant negative correlation was observed between SUZ12 protein expression and RBC count (*r* = −0.65, *p* < 0.001) and Hb level (*r* = −0.86, *p* < 0.001) (Figure 1). These results suggest that SUZ12 is involved in the clonal proliferation of PNH.

**TABLE 1:** The clinical characteristics of enrolled PNH patients.

Characteristics	Patients (*N* = 15)
Sex	8/7
Age (years)	36 (18–56)
RBC (×10^12^/L)	2.02 (0.44)
Hb (g/L)	67 (12)
WBC (×10^9^/L)	4.96 (2.10)
Platelet (×10^9^/L)	112.40 (54.10)
Ret (%)	14.34 (3.78)
LDH (U/L)	1503 (562.21)
TBIL (μmol/L)	46.57 (17.80)
IBIL (μmoul/L)	25.39 (11.08)
D‐Dimer (ng/mL)	276 (278)
FIB (mg/L)	124.2 (98.17)
CD59‐RBC (%)	52.12 (37.18)
CD59‐granulocyte (%)	85.25 (10.23)
Flaer‐monocyte (%)	69.23 (12.56)
Flaer‐granulocyte (%)	71.72 (22.16)

Abbreviations: FIB, fibrinogen; Hb, haemoglobin; IBIL, Indirect bilirubin; LDH, lactic dehydrogenase; PNH, paroxysmal nocturnal haemoglobinuria; RBC, red blood cells; Ret, reticulocyte; TBIL, total bilirubin; WBC, white blood cells.

### 3.2 MIB2 and SUZ12 Physically Interact With Each Other

**FIGURE 2:**
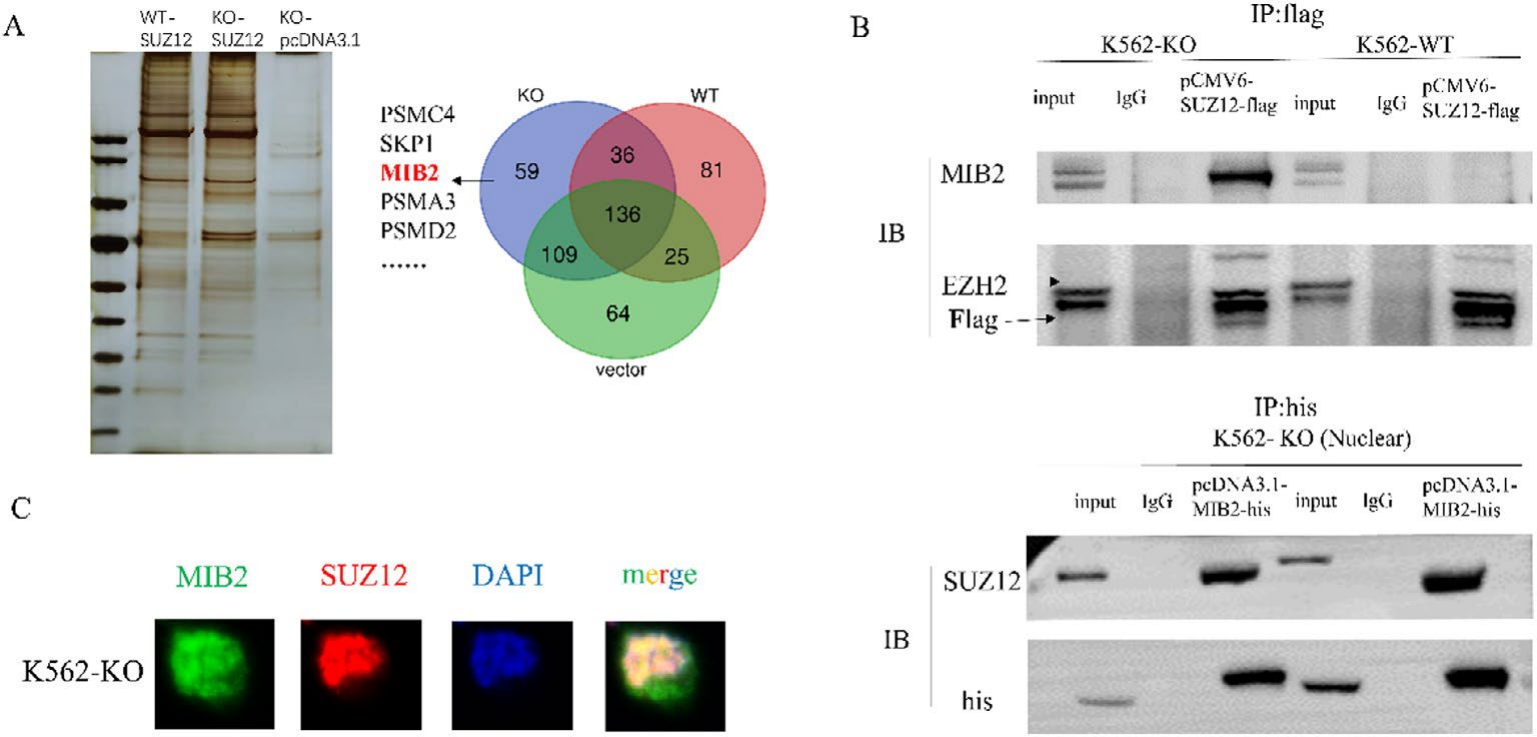
MIB2 and SUZ12 exhibit physical binding. (A) Mass spectrometry was employed to identify the physical interaction between MIB2 and SUZ12 in the K562‐WT, K562‐KO, and K562‐vector groups, aiming to determine the proteins that interact with SUZ12. (B) Immunoprecipitation experiments were performed to validate the observed physical interaction between SUZ12 and MIB2. (C) Immunofluorescence was utilised to determine the subcellular localisation of MIB2 and SUZ12.

We previously established a *PIGA* knockout K562 cell line using CRISPR/Cas9 (K562‐KO cells) as an in vitro model of PNH [6]. To identify the proteins interacting with SUZ12, we transfected SUZ12‐Flag plasmids into both K562‐KO and K562‐WT cell lines. We performed immunoprecipitation followed by mass spectrometry on FLAG‐tagged proteins to identify proteins that physically interact with SUZ12. By eliminating the pull‐down proteins from both the K562‐vector and K562‐WT groups, we identified 59 potential proteins that specifically interacted with SUZ12 in the K562‐KO cell line (Figure 2A). To validate these results, immunoprecipitation experiments were conducted after transfecting SUZ12‐Flag plasmids into both K562‐KO and K562‐WT cell lines. EZH2, a well‐known interaction partner of SUZ12 and an essential subunit of the PRC2 complex, served as a positive control in immunoblotting assays. The results revealed a physical interaction between MIB2 and SUZ12 exclusively in K562‐KO cells, whereas no such interaction was observed in K562‐WT cells. Additional validation was conducted by transfecting MIB2‐His plasmids into the K562‐KO cell line, followed by immunoprecipitation of the His‐tagged fusion protein. This process confirmed the physical binding between MIB2 and SUZ12 (Figure 2B). Immunofluorescence experiments were conducted to explore the subcellular localization of the proteins. The results indicated that the MIB2 protein was present in the cell membrane, cytoplasm, and nucleus, with co‐localization of MIB2 and SUZ12 observed in the nucleus (Figure 2C).

### 3.3 Interaction Domains Between MIB2 and SUZ12

**FIGURE 3:**
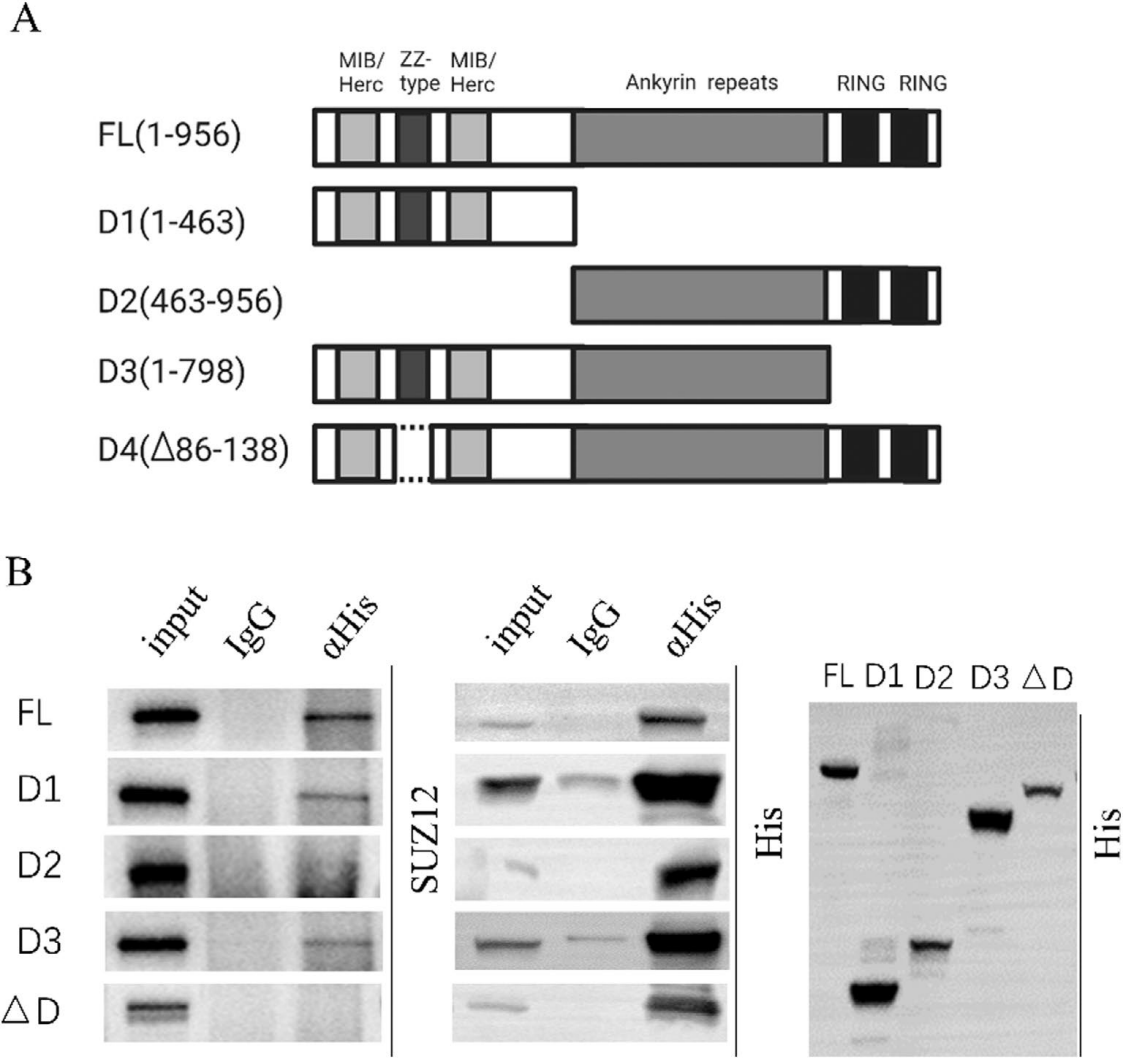
Interaction domains between MIB2 and SUZ12. (A) Mass spectrometry was employed to identify the physical interaction between MIB2 and SUZ12 in the K562‐WT, K562‐KO, and K562‐vector groups, aiming to determine the proteins that interact with SUZ12. (B) Immunoprecipitation experiments were performed to validate the observed physical interaction between SUZ12 and MIB2.

The MIB2 protein comprises a ZZ‐type domain at the amino terminus flanked by two MIB/HERC2 domains, along with several ANK repeat sequences and carboxy‐terminal RING‐type domains. To ascertain the specific interaction domain between MIB2 and SUZ12, we enzymatically digested the full‐length MIB2‐His plasmid, targeting various positions of the MIB2 domains. The plasmid was divided into the following fragments: full‐length (FL), amino acids 1–463 (D1), amino acids 463–956 (D2), amino acids 1–798 (D3), and a deletion encompassing amino acids 86–138 (ΔD) (Figure 3A). Following the transfection of MIB2‐His plasmids harbouring these distinct domain fragments into the K562‐KO cell line, we performed immunoprecipitation of the His‐tagged fusion proteins followed by immunoblotting with anti‐His and anti‐SUZ12 antibodies. The results revealed physical interactions of FL, D1, and D3 with SUZ12 (Figure 3B), while no interaction was observed between D2 and ΔD, thus emphasising the vital involvement of MIB/HERC domains and the ZZ‐type domain in the physical association between MIB2 and SUZ12.

### 3.4 MIB2 Regulates SUZ12 Ubiquitination

**FIGURE 4:**
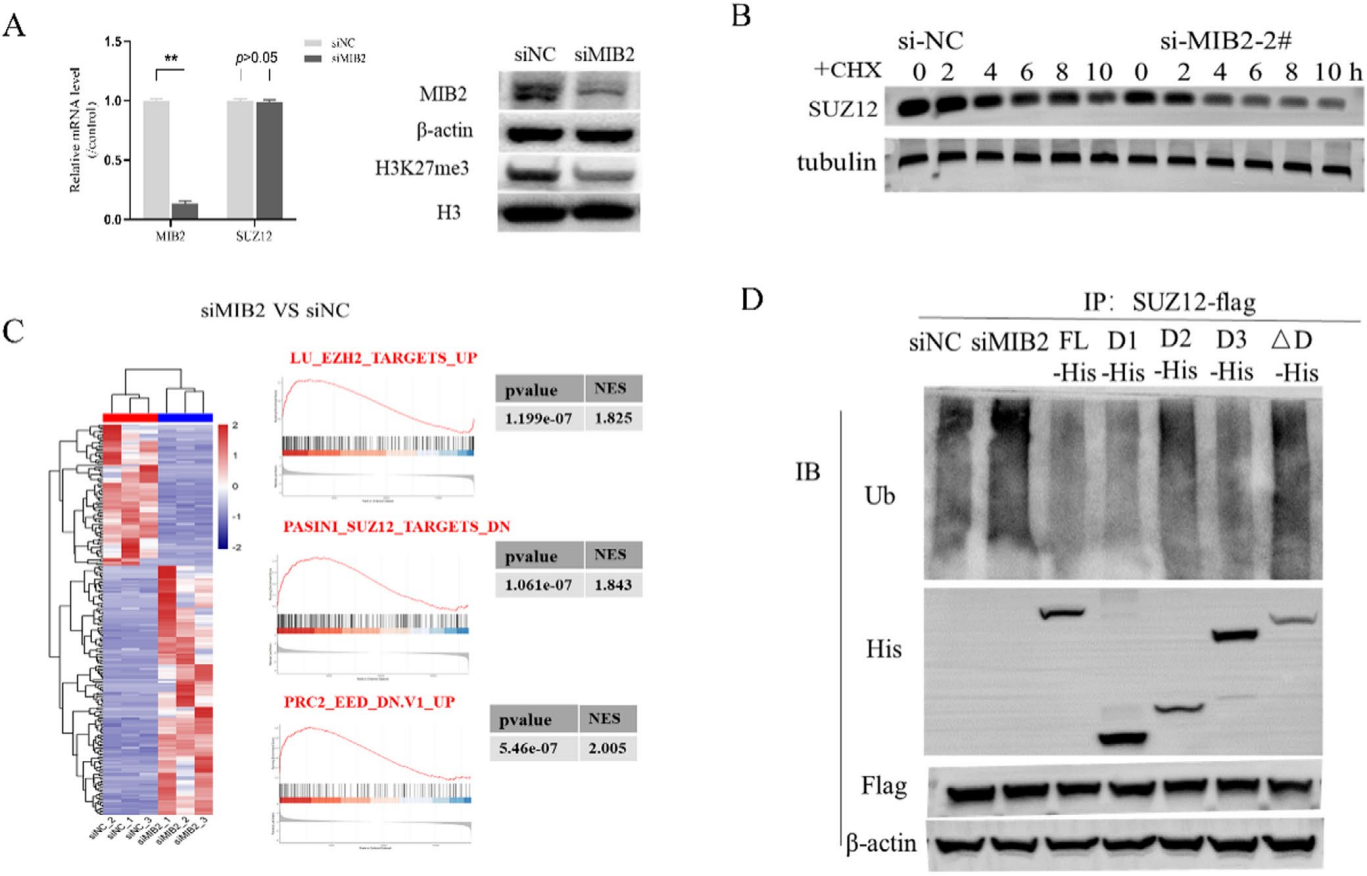
MIB2 modulates the ubiquitination level of SUZ12. (A) PCR and western blot were used to detect changes in mRNA and protein levels of cells in the siNC and siMIB2 groups. (B) CHX experiments were conducted to assess alterations in the half‐life of the SUZ12 protein between the siNC and siMIB2 groups. (C) Heatmaps were generated to present differential gene expression results and GSEA analysis findings from RNA‐seq data of the siNC and siMIB2 groups. (D) The ubiquitination level of SUZ12 was examined in the siNC group, siMIB2 group, FL group, D1 group, D2 group, D3 group, and ∆D group. **: *p* < 0.01.

Knockdown of MIB2 in the K562‐KO cell line did not significantly alter SUZ12 mRNA levels (siNC vs. siMIB2:1.00 ± 0.01 vs. 0.95 ± 0.23). However, a notable reduction in H3K27me3 levels was observed (Figure 4A). Both the siNC and siMIB2 groups were treated with cycloheximide (CHX), and protein samples were collected at various time intervals of 0, 2, 4, 6, 8, and 10 h. After the MIB2 knockdown, we observed a considerable decrease in the half‐life of SUZ12 (Figure 4B). Furthermore, RNA‐seq analysis was conducted on both the siNC and siMIB2 groups, with three biological replicates for each condition. Gene Set Enrichment Analysis (GSEA) analysis using R language revealed a notable upregulation of PRC2‐related target genes in the siMIB2 group, including DDIT3, CASP7, KLF6, and PERP (Figure 4C). Subsequently, various interventions were performed in the K562‐KO cell line encompassing the siNC, siMIB2, FL, D1, D2, D3, and D groups. Following transfection with SUZ12‐flag, SUZ12 was immunoprecipitated, followed by immunoblotting to detect ubiquitin. We observed a substantial increase in SUZ12 ubiquitination levels in the siMIB2 group compared to those in the siNC group. Moreover, compared with the FL group, the D2 and D groups exhibited a significant increase in SUZ12 ubiquitination levels (Figure 4D). These findings suggest that MIB2 knockdown enhances the ubiquitination levels of SUZ12, thereby reducing the function of the PRC2 complex.

### 3.5 MIB2 Regulates PNH Clone Proliferation by Regulating the Function of the PRC2 Complex

**FIGURE 5:**
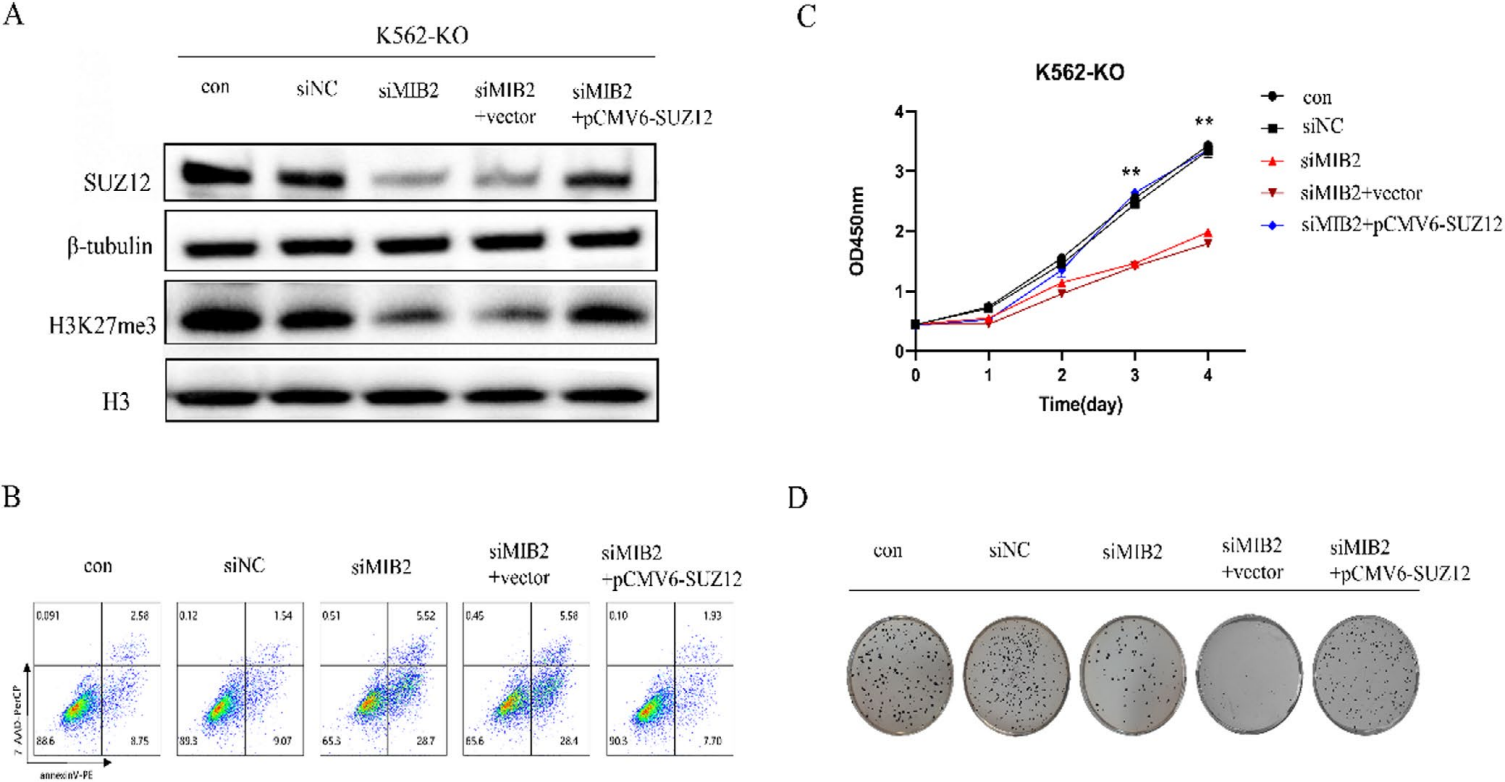
MIB2 regulates the proliferation of PNH cells. (A) Western blot was performed to investigate the changes in protein levels among the control, siNC, siMIB2, siMIB2 + vector, and siMIB2 + SUZ12 overexpression groups. (B) Flow cytometry was utilised to assess the apoptosis levels among the groups. (C) CCK‐8 assay was employed to evaluate the cell proliferation capacity among the groups. (D) Soft agar colony formation assay was conducted to measure the ability of cell colony formation among the groups.

To investigate the potential role of MIB2 in regulating PNH cell proliferation through modulation of the PRC2 complex, we conducted a series of experimental interventions and rescue assays using the K562‐KO cell line. The experimental groups included control, siNC, siMIB2, siMIB2 + vector, and siMIB2 + SUZ12 overexpression groups. Our results revealed a significant reduction in SUZ12 protein and H3K27me3 levels in the siMIB2 and siMIB2 + vector groups. Notably, when we overexpressed SUZ12 along with siMIB2 knockdown, we observed a clear restoration of both SUZ12 and H3K27me3 protein levels (Figure 5A). Moreover, flow cytometry analysis demonstrated a significant increase in apoptosis in the siMIB2 and siMIB2 + vector groups. However, in a rescue experiment, in which SUZ12 plasmid overexpression was performed simultaneously with siMIB2 knockdown, we observed a marked recovery in apoptotic levels (Figure 5B). Additionally, the CCK‐8 assay indicated a significant decrease in cell proliferation in the siMIB2 and siMIB2 + vector groups. Conversely, SUZ12 plasmid overexpression in combination with siMIB2 knockdown led to a substantial recovery in cell proliferation (Figure 5C). Furthermore, the soft agar colony formation assay demonstrated a significant reduction in colony formation ability in both the siMIB2 and siMIB2 + vector groups. In contrast, a rescue experiment involving simultaneous SUZ12 plasmid overexpression showed a notable restoration of colony formation ability (Figure 5D). Collectively, these findings strongly suggest that MIB2 plays a regulatory role in PNH clone proliferation by modulating PRC2 complex functionality.

### 3.6 The Elevation in Nuclear MIB2 Protein Level in PNH

**FIGURE 6:**
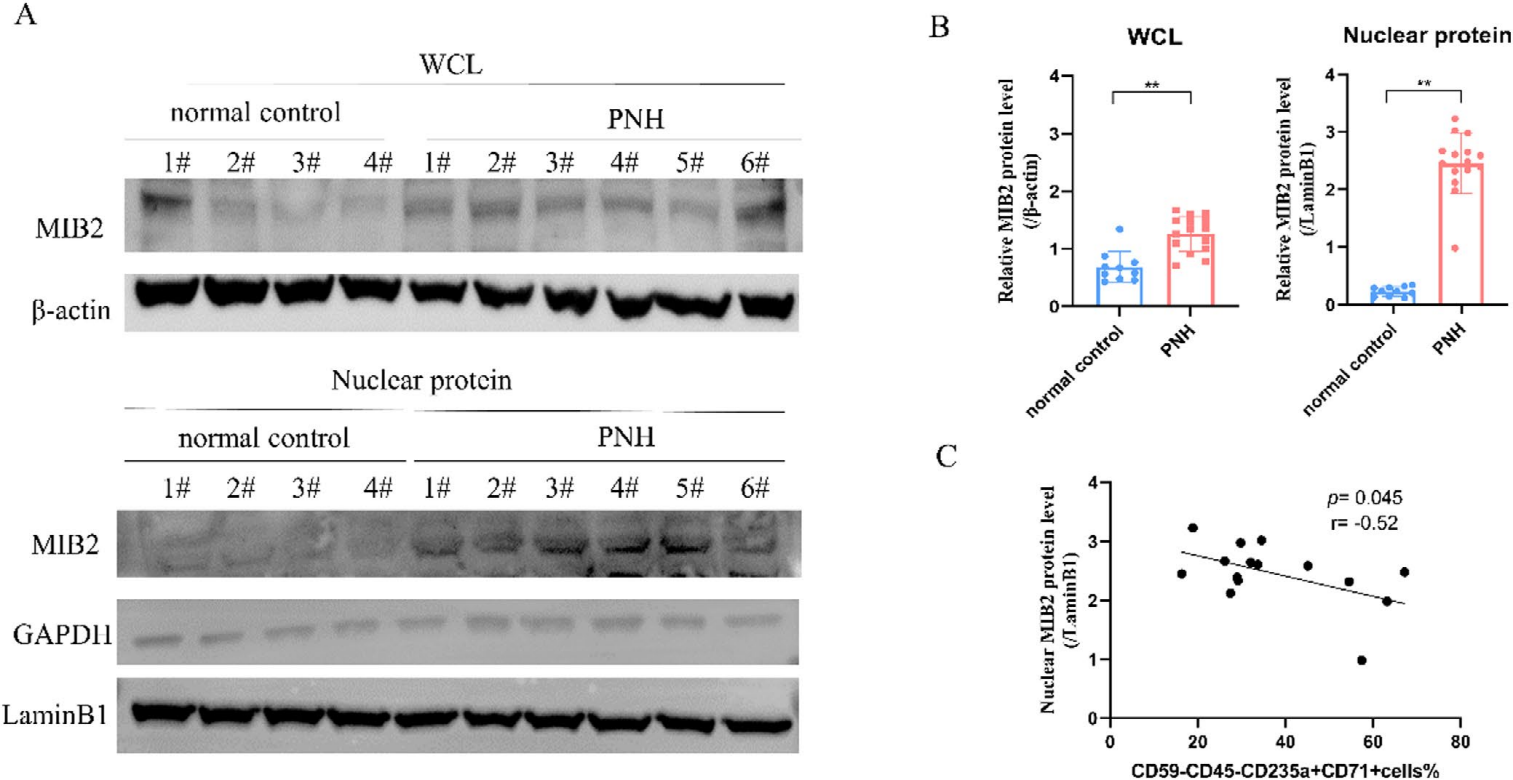
MIB2 expression levels in PNH patients. (A) Western blot was conducted to measure the protein levels of MIB2 in normal control and PNH patients, including whole cell lysates and nuclear protein extracts. (B) Graph of statistical analysis. (C) Correlation analysis between the expression level of MIB2 protein in nuclear protein extracts from PNH patients and the proportion of bone marrow CD59 negative nucleated red blood cells (**p < 0.01 compared with the control).

To further investigate the protein expression levels of MIB2 in patients with PNH, we conducted a comparative analysis with normal controls. Our findings revealed a significant up‐regulation in MIB2 protein level in both the whole‐cell lysate (1.26 ± 0.30 vs. 0.68 ± 0.27, *p* < 0.0001) and nuclear protein lysate (2.45 ± 0.52 vs. 0.23 ± 0.08, *p* < 0.0001) in PNH patients, particularly in the nuclear fraction (Figure 6A,B). Moreover, we explored the correlation between nuclear MIB2 protein expression and the proportion of CD59‐nucleated red blood cells in the bone marrow of patients with PNH. The results demonstrated a clear negative correlation between nuclear MIB2 protein expression levels and the percentage of CD59‐nucleated red blood cells in the bone marrow (*r* = −0.52, *p* = 0.045) (Figure 6C). These findings strongly suggest an elevation in nuclear MIB2 protein levels in PNH and its association with the proportion of PNH clones.

**FIGURE 7:**
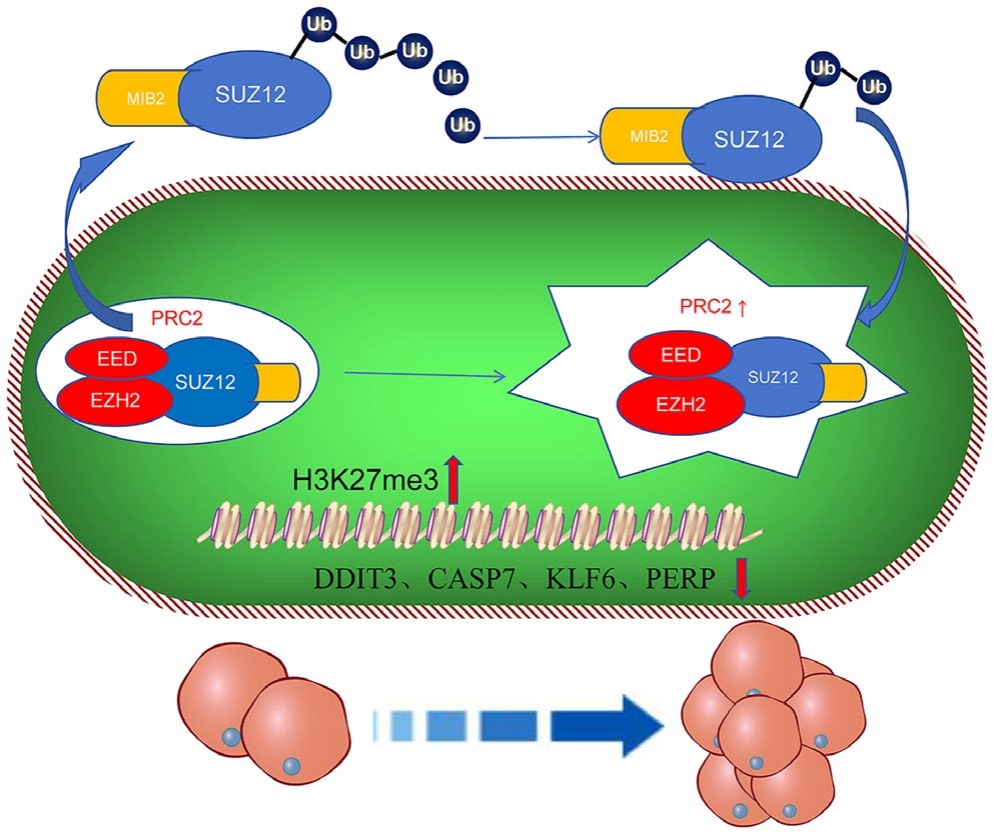
Summary of the mechanisms underlying MIB2 and SUZ12 mediated PNH clone proliferation. MIB2 binds to SUZ12 and leads to the activation of the PRC2 complex and downstream H3K27m3 methylation by affecting its ubiquitination level. The expression of downstream genes, including DDIT3, CASP7, KLF6, and PERP, decreases, which induces the proliferation of the PNH clones.

In our study, we confirmed that in PNH clones, MIB2 binds to SUZ12 and leads to the activation of the PRC2 complex and downstream H3K27m3 methylation by affecting its ubiquitination level. The expression of downstream genes, including DDIT3, CASP7, KLF6, and PERP, decreases, which induces the proliferation of the PNH clones (Figure 7).

## 4 Discussion

Epigenetics is a prominent area of research in the field of tumour development, and the polycomb group protein (PcG) family has emerged as a crucial set of epigenetic regulatory molecules [10]. PcG proteins play vital roles in diverse physiological and pathological processes, including cell proliferation, differentiation, and tumorigenesis [11, 12, 13]. SUZ12, in conjunction with EED and EZH2, constitutes the PRC2 complex. As an essential subunit of PRC2, it facilitates the trimethylation of histone H3 at lysine 27, resulting in a condensed chromatin configuration that suppresses the transcriptional activity of RNA polymerase II [14, 15]. This process exerts a critical influence on PRC2‐mediated post‐transcriptional gene silencing and subsequently participates in the regulation of diverse biological processes, including cell proliferation, haematopoietic cell differentiation, and development. Among them, SUZ12 is considered an important oncogene involved in several cancers such as bladder cancer, non‐small cell lung cancer, and ovarian cancer, and is currently a research hotspot in targeted therapy for various types of tumours [16, 17, 18].

Our previous studies confirmed the upregulation of SUZ12 expression in aberrant clones among patients with PNH. This increased expression modulates various cellular processes, including cell proliferation, apoptosis, and the cell cycle, by modulating H3K27me3 levels, ultimately leading to the expansion of PNH clones. Further studies are required to ascertain whether specific proteins within PNH cells can regulate SUZ12 levels and subsequently modulate the functionality of the PRC2 complex. Some researchers have reported that SUZ12 contains ubiquitination modification sites and is regulated by the ubiquitin‐proteasome system through its interaction with USP3 as a substrate [8]. Consequently, we focused on ubiquitin/deubiquitinase enzymes. Ubiquitination is a post‐translational modification of proteins that is involved in an energy‐dependent protein degradation mechanism via the action of the proteasome [19]. It is well known for its involvement in the regulation of various biological phenomena, including the cell cycle, signal transduction pathways, and transcription [20, 21].

Building upon prior research, this study investigated the presence of potentially specific proteins within cells exhibiting PIGA knockouts. These proteins modulate the function of the PRC2 complex by directly influencing SUZ12 protein levels, consequently affecting cell proliferation. Using a proteomics‐based methodology, we successfully identified MIB2 as a novel interacting protein of SUZ12. As an E3 Ubiquitin ligase, MIB2 plays a key role in several physiological activities and participates in NFκB, Notch, apoptosis, Ferroptosis, and many other cell signalling pathways [22, 23, 24, 25, 26]. Structurally, MIB2 comprises an N‐terminal ZZ‐type domain flanked by two MIB/HERC2 domains, followed by several ANK repeat sequences, and it terminates with two C‐terminal RING‐type domains. Immunofluorescence experiments were performed to elucidate the specific interaction domains between SUZ12 and MIB2, which revealed a direct association between the MIB/HERC2 domains and the ZZ‐type domain of SUZ12. Other studies have indirectly confirmed our findings. Shi et al. discovered that the ZZ‐type domain possesses a histone recognition module that enables it to recognise the H3 tail. Using the ZZ domain as a pull‐down tool, they identified nine proteins, including MIB2 and MIB1, that harbour the ZZ domain and bind to the H3 tail [27, 28]. As an E3 ligase, MIB2 mediates the ubiquitination of delta receptors, which serve as Notch ligands. By ubiquitinating the intracellular domain of Delta, MIB2 actively regulates the Notch signalling pathway mediated by delta, leading to endocytosis of the delta receptor and modulation of its activation of Notch signalling [22, 29]. Zhang et al. reported that MIB2 ubiquitinates and activates DLL3 on the oocyte membrane, leading to the binding and activation of Notch2, and ultimately resulting in the cleavage of NICD2. NICD2 directly activates AKT in the cytoplasm, thereby regulating oocyte meiosis and quality [30]. Vanessa et al. discovered that the MIB/HERC2 domain of MIB2 regulates IKK ubiquitination, leading to the recruitment and activation of TAK1. This process controls the activation of NFκB mediated by BCL‐10 [31].

Our study identified a regulatory role of MIB2 in the ubiquitination of substrate proteins. Unexpectedly, the siMIB2 group exhibited increased ubiquitination of its substrate protein, SUZ12, contradicting the anticipated outcomes. Furthermore, the siMIB2 group displayed reduced levels of H3K27me3, and RNA‐seq analysis confirmed the activation of the PRC2 target gene set, providing additional evidence to substantiate the reliability of these findings. The interaction between SUZ12 and MIB2 may involve other proteins or regulatory factors, necessitating further investigation.

In summary, MIB2 plays a crucial role in regulating PNH clone proliferation by regulating the ubiquitination levels of SUZ12 and modulating the function of the PRC2 complex. Thus, MIB2 may be a novel therapeutic target for PNH.

## Author Contributions

**Hui Liu:** data curation (equal), investigation (equal), project administration (equal), validation (equal), writing – original draft (equal). **Lijie Zeng:** data curation (equal), formal analysis (equal), methodology (equal), resources (equal). **Chaomeng Wang:** methodology (equal), resources (equal), software (equal). **Yingying Chen:** formal analysis (equal), investigation (equal), methodology (equal), resources (equal). **Liyan Li:** data curation (equal), methodology (equal), project administration (equal), validation (equal). **Zhaoyun Liu:** conceptualization (equal), formal analysis (equal), software (equal), supervision (equal), visualization (equal). **Honglei Wang:** methodology (equal), visualization (equal). **Rong Fu:** conceptualization (equal), funding acquisition (lead), writing – review and editing (lead).

## Conflicts of Interest

The authors declare no conflicts of interest.

## Data Availability

The data presented in this study are available on request from the corresponding author.
